# Modeling the Chronic Loss of Optic Nerve Axons and the Effects on the Retinal Nerve Fiber Layer Structure in Primary Disorder of Myelin

**DOI:** 10.1167/iovs.16-19871

**Published:** 2016-09-19

**Authors:** Leandro B. C. Teixeira, James N. Ver Hoeve, Joshua A. Mayer, Richard R. Dubielzig, Chelsey M. Smith, Abigail B. Radcliff, Ian D. Duncan

**Affiliations:** 1 Department of Pathobiological Sciences School of Veterinary Medicine, University of Wisconsin-Madison, Madison, Wisconsin, United States; 2 Department of Ophthalmology and Visual Sciences, School of Medicine and Public Health, University of Wisconsin-Madison, Madison, Wisconsin, United States; 3 Department of Medical Sciences, School of Veterinary Medicine, University of Wisconsin-Madison, Madison, Wisconsin, United States

**Keywords:** optic nerve, OCT, retina

## Abstract

**Purpose:**

We determined whether the chronic lack of optic nerve myelination and subsequent axon loss is associated with optical coherence tomography (OCT) changes in the retinal nerve fiber layer (RNFL), and whether this models what occurs in multiple sclerosis (MS) and confers its use as a surrogate marker for axon degeneration.

**Methods:**

Using an animal model of Pelizaeus-Merzbacher disease (*shp*) bilateral longitudinal measurements of the peripapillary RNFL (spectral-domain OCT), electroretinograms (ERG), and visual evoked potentials (VEP) were performed in affected and control animals from 5 months to 2 years and in individual animals at single time points. Light and electron microscopy of the optic nerve and retina and histomorphometric measurements of the RNFL were compared to OCT data.

**Results:**

Of the *shp* animals, 17% had an average reduction of OCT RNFL thickness on the superior retinal quadrant compared to controls (*P* < 0.05). Electroretinograms showed normal photopic A- and B-waves but flash VEPs were disorganized in *shp* animals. Morphologically, the *shp* retinas and optic nerves revealed significant RNFL thinning (*P* < 0.001) without retinal ganglion cell (RGC) loss, decrease total and relative retinal axonal area, and loss of optic nerve axons. There was strong positive correlation between OCT and morphometric RNFL thickness measurements (*r* = 0.878, *P* = 0.004).

**Conclusion:**

The loss of optic nerve axons demonstrated in the *shp* model resulted in moderate thinning of the RNFL confirmed by OCT and histology. These results indicate that OCT-derived RNFL measurement can be a useful surrogate biomarker of optic nerve axon loss and potentially disease progression in demyelinating diseases.

The finding of retinal nerve fiber layer (RNFL) atrophy in patients with multiple sclerosis (MS) has received much attention and has been proposed to reflect axon loss in the visual pathway,^1–6^ and is potentially indicative of more general axonal pathology in the central nervous system (CNS).^7^ In these studies, RNFL thickness was assessed noninvasively using optical coherence tomography (OCT). Thinning of the RNFL generally is associated with loss of axons in the optic nerve, but thinning also could result from a decrease in axon diameter or changes in surrounding retinal glia. A detailed study of the histopathology of the retina in 82 MS patients showed substantial loss of retinal ganglion cells (RGCs) associated with perivascular inflammation and gliosis, suggesting that a primary loss of RGCs also may result in axon loss and RNFL atrophy.^8^ While studies in experimental glaucoma have correlated optic nerve axon loss with RNFL atrophy measured by OCT,^9,10^ this information is lacking in MS or its experimental models where myelin sheath loss or absence is the primary defect.

A complete understanding of the significance of RNFL thinning as a surrogate marker of axon loss in MS is a high research priority,^11,12^ and a critical issue for the use of OCT as a putative surrogate marker of axon loss in MS and other degenerative CNS disorders. In 2007, an expert panel consensus report proposed that an animal model might answer the other question as to whether there is a direct correlation between axon loss in the CNS and RNFL atrophy.^13^ Mouse^14^ and rat^15^ experimental autoimmune encephalomyelitis models showed that inflammation in the optic nerve could lead to RGC death but no OCT nor RNFL measurements were performed in either study. We investigated this issue using a canine model (shaking pup - *shp*) that presents a genetic myelin disorder associated with a mutation in the proteolipid protein (*PLP1*) gene, allelic to Pelizaeus-Merzbacher disease (PMD), and that develops a chronic axon loss in the optic nerve, similar to the loss of demyelinated axons seen in MS patients.^16^ This model is particularly well suited to address this question because it is a disorder of dysmyelination without inflammation, and axons in the optic nerve predominantly lack a myelin sheath. Thus, loss of axons without or with reduced inflammation mimics that seen in progressive MS^17,18^ and should provide a relatively uncontaminated estimate of change in OCT-measured RNFL thickness attributable to axon loss in the nerve.

We used spectral domain OCT (sdOCT) to quantitate the RNFL thickness longitudinally in a group of dogs and at single time points in others. We also recorded visual evoked cortical potentials (VEPs) and electroretinograms (ERGs). These data then were compared to histomorphometric measurements of the RNFL thickness and axon density. Analyses of these data were aimed at determining whether the loss of axons within the optic nerve led to thinning of the RNFL, identified in vivo by OCT, and if the OCT RNFL measurement correlated with quantitative histology.

## Materials and Methods

### Animals

The *shp* mutant is maintained in a colony at the University of Wisconsin-Madison. The *shp* mutation arose in a line of Welsh Springer spaniel dogs.^16,19,20^ Affected male dogs and female carriers were identified by PCR of genomic DNA isolated from whole blood.^21^ All animals were handled and treated according to the ARVO Statement for the Use of Animals in Ophthalmic and Vision Research and the guidelines and recommendations of the Research Animal Resources Center and the Animal Care and Use Committee at the University of Wisconsin-Madison.

### OCT and VEPs

A total of 12 dogs underwent sdOCT scans. All subjects were male Welsh Springer spaniels between 3 months and 2.5 years old. Shaking pups (*shp*s) were identified by their obvious tremor phenotype^16^ and confirmed by PCR. Four dogs from the same litter, two *shp*s and two wild type control males, were scanned on five separate occasions, beginning at 3 months of age then approximately every 3 to 5 months, over the course of 1 year and 7 months. The remaining eight subjects were each tested once at the time of euthanasia at multiple time points (*shp*s, at 4 mo [*n* = 1], 1 year [*n* = 2], and 2 years [*n* = 2] and controls, 4 months [*n* = 2], 2 years [*n* = 1]).

All dogs were premedicated with dexmedetomidine, anesthetized with isoflurane, and ventilated under the care of a veterinary anesthesiologist. Pupils were dilated with phenylephrine 1% and tropicamide 2.5% drops. Optical coherence tomography was performed using a Cirrus SD-OCT (Software version 5.9.1.096; Carl Zeiss Meditec, Dublin, CA, USA). Retinal nerve fiber layer thickness measurements were obtained from software supplied by the manufacturer. Each scan was evaluated for accuracy of segmentation and measurements verified using software calipers. Measures of neuroretinal rim thickness (NRT), disk area, average cup-to-disc (C/D) ratio, and cup volume were obtained from the scans. Measures of the optic nerve head were obtained using Cirrus software, which defined the inner cup as the termination of Bruch's membrane within the 6 × 6 mm cube scan and then finds the shortest distance vertically to the inner limiting membrane (ILM).^22^ This allowed consistent centering of the 3.4-mm circle used for RNFL measurement and the edge of the neural rim. Specific parameters calculated by the Cirrus SD-OCT were used in the analysis and include neural rim area, optic disc area, average C/D ratio (average of 180 radial line measurements around the optic nerve cup), and optic cup volume. Retinal nerve fiber layer thickness was measured as the distance between the ILM and the border of the RNFL with the ganglion cell layer. Cirrus software plotted the estimated RNFL thickness as a function of clock-hours around the nerve head and gave average thickness values for the temporal, superior, nasal, and inferior quadrants.

Electroretinograms and VEPs were obtained at the same session at OCT measurements. Electroretinograms were recorded with a GoldLens contact lens electrode and VEPs with subdermal electrodes using an Espion 2 Color-burst system (Diagnosis, LLC, Loewell, MA, USA). Flashes of cd-s m^−2^ were delivered at a rate of 2/sec and an average of 80 flashes were obtained.

### Light and Transmission Electron Microscopy (TEM)

Control and *shp* dogs were deeply anesthetized with isoflurane followed by intracardiac perfusion with 0.01 M PBS (pH 7.4) followed with weak and strong Karnovsky's fixative.^19^ The right and left globes were immersion-fixed in 2.5% glutaraldehyde in 0.1 M PBS (pH 7.4) at 4°C overnight. A 2-mm long cross-section of the retina was sampled in four quadrants (dorsal, ventral, temporal, and nasal), 1 mm away from the periphery of the optic nerve to match the areas of OCT measurement. A segment of the mid optic nerve was trimmed and the retinal and optic nerve samples processed for epon embedding.^19^ Sections (1 μm) were stained with toluidine blue and examined under the light microscope. Ultrathin sections (90 nm) of retina and optic nerve were analyzed using a JEOL-100CX electron microscope (JEOL Ltd., Tokyo, Japan). In addition to the optic nerves from the *shp*s that were studied by OCT, we examine optic nerves from other *shp*s including six over 2 years of age (Duncan, unpublished data).

### Image Quantification

#### RNFL Thickness and Ganglion Cell Density – Light Microscopy.

To quantify the RNFL thickness histologically, plastic-embedded, toluidine blue-stained 1-μm cross-sections of the superior retina of both globes were imaged using a bright light microscope Olympus BX43 (Olympus, Inc., Center Valley, PA, USA) with attached Olympus DP72 digital camera (Olympus, Inc.), and captured using commercially available image-analysis software (cellSens Dimension; Olympus, Inc.). Consecutive and nonoverlapping high-magnification (×100) digital images, covering the whole retina sample, were acquired and 130 to 160 equidistant RNFL thickness measurements were taken with the help of the image-analysis software. Using the same images the number of RGCs present in the plane of section were counted and the total RNFL area was measured to calculate the RGC density (RGCs/mm^2^).

#### RNFL Axon Quantitation – Ultrastructure.

Ultrathin (90 nm) cross-sections of the retinas were analyzed by TEM. A total of 20 nonoverlapping high-magnification (×8500) digital images of the superior retina of both globes was taken. Transmission electron microscopic images were processed and analyzed using ImageJ software (available in the public domain at http://imagej.nih.gov/ij/ by the National Institutes of Health, Bethesda, MD, USA). Briefly, the image's scale bar was used to set up the system's spatial calibration. Using the “threshold tool” and “freehand tool,” the axons in the image were selected, and the total axonal area (sum of the area of all axons measured) and the percentage of axonal area (total axonal area*100/total image area) were measured.

### Statistical Analysis

A Pearson product-moment correlation coefficient was computed to assess the relationship between the OCT RNFL thickness, histologic RNFL thickness and percentage of axonal area in the retina.

Two-tailed Student's *t*-tests were performed to compare histologic RNFL thickness, RGCs density, retinal total axonal area, retinal percentage of axonal area, and distribution of retinal axonal diameter between control and affected animals at the same time points. Additionally two-sample ANOVAs were used to calculate the differences in OCT and VEP measurements. *P* values < 0.05 were deemed significant. All statistical analyses were performed with Excel (Microsoft Corporation, Redmond, WA, USA), R (available in the public domain at https://www.r-project.org), or Prism (GraphPad Software, Inc., La Jolla, CA, USA) software.

## Results

### Establishing the Model of Chronic Nerve Fiber Loss in the Optic Nerve

**Figure 1:**
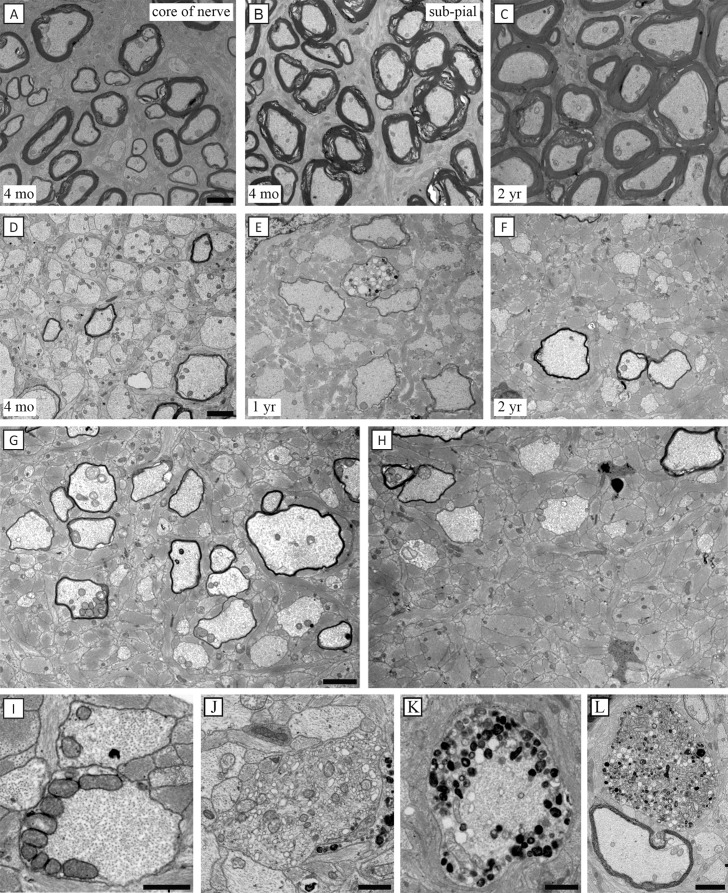
Axon loss over time in the *shp* optic nerve. The optic nerves of controls at 4 months and 2 years of age showed almost complete myelination of all axons (**A**–**C**), except in the core of the nerve at 4 months of age where occasional small diameter axons were nonmyelinated (**A**). In the *shp* animals, at 4 months of age, the axon density appeared normal, but there was a gradual loss of axons from 4 months to 2 years of age with subsequent gliosis (**D**–**F**). At 2 years of age in the same optic nerve, an area with thinly myelinated and nonmyelinated axons (**G**) was adjacent to a highly gliotic area with severe axon loss (**H**). A range of axonal changes was seen in the optic nerves at all time points though such axons were not frequent (**I**–**L**). Scattered axons were noted to have a subaxolemmal increase in mitochondria (**I**). Other axons were noted to have an increase in vesicular profiles (**J**), dense bodies in the subaxolemmal space (**K**) or the entire axon (**L**). *Scale bars*: 2 μm (**A**–**E**, **I**); 1 μm (**F**–**H**)

We examined the optic nerves (Figs. 1A–C) from controls at intervals from 4 months to 2 years and from *shp*s from 3 months to 2.5 years of age (Figs. 1D–L) to determine whether there was qualitative evidence of axon loss and whether this was progressive. At 3 to 4 months, axon density in *shp*s appeared normal compared to age-matched controls, though, as expected, the majority of axons lacked a myelin sheath (Fig. 1D). By 12 months, scattered optic nerve areas showed a decrease in axon density and an increase in astrocytes numbers and density (gliosis) (Fig. 1E). By two years of age, the axon loss was more obvious and gliosis more prominent (Fig. 1F). However, the loss of axons throughout the nerve was uneven and in the same nerve, adjacent areas showed a diffuse axon loss (Fig. 1G) or resembled a glial scar (Fig. 1H). This fact rendered axon quantitation unreliable and the nerve was too large to consider total axon counts at the EM level. Despite the uneven distribution, the areas of greatest axon loss were in the center of the nerve, which is dominated by small diameter myelinated axons, suggesting that this caliber of fibers was at greatest risk.

While this loss of axons appeared chronic, degenerative axonal changes were identified at all time points examined. The main axonal lesions noted were an increase in mitochondria, usually subaxolemmal (Fig. 1I); abnormal collection of organelles, often lysosomes or dense bodies; axonal swelling, and rarely cytoplasmic tubulovesicular profiles (Figs. 1J–L). These abnormal axons were scattered and infrequent; however, often only one abnormal axon was observed per electron microscopic (EM) grid square (103 μm^2^). It can be concluded that all *shp*s over 2 years of age have axon loss in the optic nerve as confirmed in six other cases over 2 years of age (Duncan, unpublished data).

### OCT Measurements of the Retina, ERG and VEPs

Retinal nerve fiber layer scans from affected and unaffected dogs were evaluated for reliability and repeatability. The coefficient of variation (COV) of global average RNFL thickness across the four test sessions was 8.2% and 8.9% for the two affected dogs and 2.2% and 8.2% for the unaffected dogs. These values compared favorably with the COVs from human studies of RNFL thickness^23–25^ despite that facts that most human studies are based on multiple measurements made during a single visit, the *N* for this study is very small, and there is a strong likelihood that there was continued change in RNFL in the dogs across the 2-year period. The intraclass correlation (ICC) for global RNFL, while good at 0.65, is somewhat lower than is typically reported in studies of humans that typically have very large *N*s and short intertest intervals.

**Figure 2:**
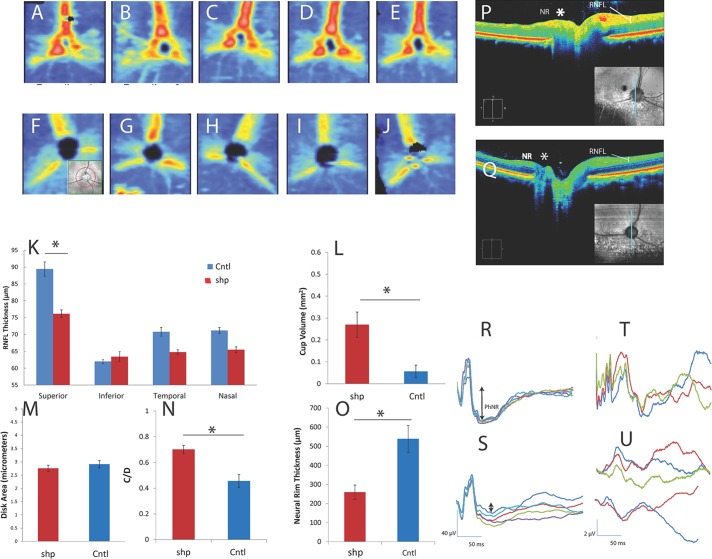
The RNFL of the *shp* dogs was thinner than that of controls as shown by OCT with normal ERGs and abnormal VEPs. Retinal nerve fiber layer thickness maps obtained at approximately 6-month intervals starting at 4 months of age from an unaffected (**A**–**E**) and *shp* (**F**–**J**) littermates. Note thicker (*red*) RNFL in superior retina of the unaffected animal compared to the *shp* animal. *Inset* in (**F**) shows the location of the circular scan used for quantification by Cirrus software. There was potential OCT evidence of progressive loss of RNFL in the *shp* dogs taken after the first scan at 4 months of age, at which time RNFL development was not yet complete; some loss of signal intensity is noted in the superior region between 10 months (**G**) and the final scans at approximately 24 months (**J**). Comparing all unaffected and *shp*s dogs using the last measurement before euthanasia, RNFL measurements from standard circular scans (*inset*, **F**) were significantly thinner in the *shp* dogs in the superior quadrant (**K**). The volume of the optic cup (**L**) was markedly larger in *shp* compared to unaffected dogs (*P* < 0.01), although there were no differences in the area of the optic nerve head disk (**M**). The C/D ratio (**N**) also was significantly increased in *shp* dogs (*P* < 0.05). Neural rim tissue around the optic nerve head also was significantly thinner (*P* < 0.01) in *shp* compared to control (**O**) animals. High-resolution raster scans through the optic nerve head of an unaffected dog demonstrated a robust neural rim (*) and shallow cup (**P**). In contrast, *shp* dogs (**Q**) had markedly reduced NRT (*NR*), thinner RNFL, and deep excavated cups. The *vertical line* shown in the *insets* in (**P**) and (**Q**) represent the areas of the fundus sampled on OCT. Photopic full-field ERGs also were robust in normal (**R**) and *shp* (**S**) dogs, indicating normal photoreceptor and bipolar function. Flash VEPs in normal dogs typically contained an early (<50 ms) oscillatory wavelets followed by a large positive voltage waveform. Compared to ERGs, VEPs were markedly reduced in amplitude in affected dogs (**U**) and were devoid of early oscillatory wavelets seen in unaffected littermates (**T**).

The average sdOCT-determined RNFL thickness from the circular scan from all seven *shp* dogs was 66.14 ± 4.67 μm compared to the average RNFL thickness of 74.11 ± 3.99 μm in the five unaffected dog eyes (using the averages of the right and left eyes from the final scans of the four longitudinally tested dogs). However, the standard Cirrus software optic disk cube quadrant analysis revealed marked differences in RNFL thickness by quadrant in normal and *shp* dogs. For control and *shp* dogs, the superior quadrant RNFL was thicker than the inferior quadrant, and the nasal and temporal quadrants were of intermediate thickness (Figs. 2A–J). A mixed-design ANOVA indicated that groups differed significantly by quadrant (*P* < 0.05) and a planned comparison indicated that this difference reached statistical significance for the superior quadrant. The average RNFL thickness in the superior quadrant was 89.44 ± 2.12 μm in controls compared to 76.15 ± 1.19 μm in *shp* dogs, or a difference of 17% (Fig. 2K). There were no differences in the area of the retina occupied by optic nerve head disk, yet the volume of the optic cup was markedly larger in *shp* compared to unaffected dogs, as determined by automated measurements from the Cirrus circular optic nerve head scans (Figs. 2L–M). The C/D ratio also was significantly larger in the *shp* (Fig. 2N). The NRT also was significantly thinner in *shp* compared to controls (Fig. 2O).

Electroretinography revealed normal Photopic A and B waves in control and *shp* dogs (Figs. 2R–S). However, the late Photopic negative response (PhNR) was blunted in *shp* dogs (Fig. 2S). The flash-evoked visual evoked potential (fVEP) was markedly abnormal in affected animals tested at the youngest age with possibly some deterioration in retinocortical transmission over the 2-year test period (Fig. 2U).

### RNFL Thickness, Retinal Axonal Quantification, and RGC Density

**Figure 3:**
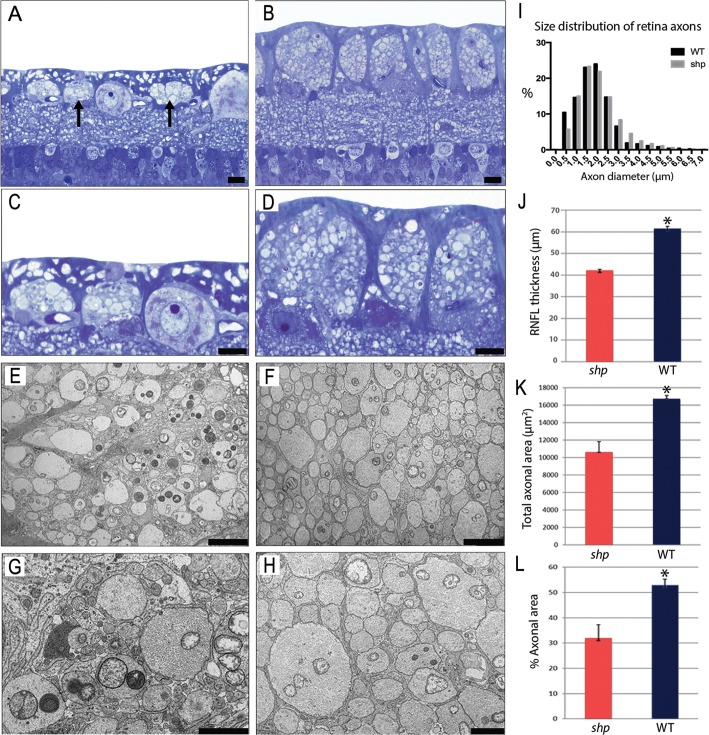
There was qualitative and quantitative evidence of loss of axons in the RNFL of *shp* dogs. The RNFL in *shp* dogs (**A**, *arrows*) was clearly thinner than in control (**B**) dogs. This difference is more pronounced in the higher magnification images (**C**, **D**). Morphometric measurements of the superior retina showed significant (*P* < 0.001) thinning of the RNFL in *shp* compared to control (**J**) dogs. Normal-appearing RGCs were prominent in the *shp* retina. There were no differences of note in the fiber diameter spectra of the axons in the RNFL between control and the *shp* (**I**) animals. Ultrastructurally, the RNFL of the *shp* dogs showed greater spacing between axons with denser neuropil (**E**, **G**) and had occasional swollen axons with increased numbers of irregular mitochondria when compared to control dogs (**F**, **H**). Morphometric data showed that *shp* dogs had a significant (*P* < 0.005) decreased in total axonal area (sum of the area of all axons measured, **K**) and percentage of axonal area (total axonal area*100/total image area; **L**). (**A**–**D**) Toluidine blue stain. (**E**–**H**) TEM. *Scale bars*: 20 μm (**A**, **B**), 10 μm (**C**, **D**), 5 μm (**E**, **F**), 2 μm (**G**, **H**).

Clear differences were seen in 1-μm sections between the control and *shp* RNFLs, especially in the superior quadrant (Figs. 3A–D). Morphometric measurements of the superior retina, obtained from approximately the same location as the sdOCT scans, showed significant (*P* < 0.001) thinning of the RNFL in *shp* compared to control dogs (Fig. 3J). However, there were no significant differences in the RGC density between *shp* (5143 ± 95 RGCs/mm^2^) and control (5242 ± 218 RGCs/mm^2^) dogs in the measured regions.

Morphometric data of the electron micrographs of the retina showed that *shp* dogs had a significant (*P* < 0.005) decrease in the total axonal area (sum of the area of all axons measured) (Fig. 3K) and the percent of axonal area (total axonal area*100/total image area; Fig. 3L). The measurement of axon diameters revealed no significant differences in the distribution of axonal size throughout the retina between *shp* and control dogs (*P* > 0.999; Fig. 3I). There was a strong positive correlation between OCT RNFL thickness and histologic RNFL thickness (*r* = 0.878, *P* = 0.004) and between OCT RNFL thickness and percentage of axonal area in the retina (*r* = 0.788, *P* = 0.02). The ultrastructural analysis of the *shp* and control retinas demonstrated a clear difference between the two. In controls, nonmyelinated axons of variable diameter were closely packed with thin astrocyte processes interdigitating between them (Figs. 3F, 3H). In contrast, in the *shp* retina the density of nonmyelinated axons in the RNFL was decreased and astrocyte processes and other cellular elements (such as Müller cell processes) were more prominent (Figs. 3E, 3G). Mitochondria were more numerous in some axons and some appeared swollen, yet the latter may have been due to fixation as this also was seen, but to a lesser extent, in the control RNFL.

### Optic Nerve Microscopy in Dogs Undergoing OCT

**Figure 4:**
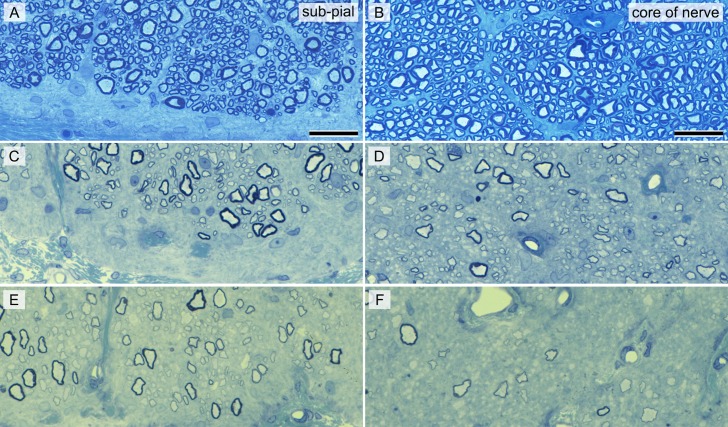
There was qualitative evidence of axon loss in the optic nerve in *shp* dogs undergoing longitudinal OCT recording. Sections 1 μm from the subpial (**A**–**E**) and core (**B**–**F**) of the optic nerves of a control (**A**, **B**) and the two *shp*s (**C**–**F**) that were monitored over 20 months. Note the lack of myelinated axons in the *shp* dogs, though one of the two dogs (**E**, **F**) showed a greater lack of myelinated axons. While there appeared to be a severe and generalized lack of axons in the core of the optic nerve in one *shp* animal, there still were many small nonmyelinated axons that could not be resolved at this magnification (**F**) but were seen on EM. Toluidine blue stain. *Scale bars*: 20 μm.

There was a clear difference in the numbers of myelinated axons in the 2-year-old control (Figs. 4A, 4B) compared to the 2-year-old *shp* (Figs. 4C–F) optic nerves as seen in the 1-μm sections. This also was the case in all other *shp*s at 2 years of age and older. The lack of axons was more prominent in one of the two *shp*s dogs that were evaluated longitudinally by OCT (Figs. 4E–F), especially in the center of the nerve (Fig. 4F). In these two animals there also were markedly fewer myelinated axons than in controls and an apparent, mild diffuse decrease in axons. On EM, however, many small diameter nonmyelinated axons were present that were not detected by light microscopy, and only in randomly scattered areas, especially the middle of the nerve, areas of loss of axons and pronounced gliosis were seen (Data not shown).

## Discussion

The present data provide strong evidence that optic nerve axon loss leads to subsequent RNFL atrophy that can be detected by OCT in the *shp* mutant, a model of Pelizaeus-Merzbacher disease that presents a slow and partial loss of axons in the optic nerve.^16^ Spectral domain OCT RNFL thickness measurements of the superior retina of the *shp* dogs showed significant thinning that was confirmed by histomorphometry of light and EM images. The optic nerves showed evidence of axon degeneration from early time points with subsequent axon loss scattered throughout the nerve ranging from mild to severe with marked gliosis.

Measurement of the RNFL thickness as a surrogate marker of axon loss in the optic nerve of MS patients has become a topic of major interest and importance. From the original reports of RNFL thinning in MS,^4,6,26,27^ it has now become the subject of intense investigation and has been proposed to be used as an outcome measure in many MS clinical trials.^12^ However, to our knowledge there have been no reports that compared sdOCT RNFL measurements with the pathology of the retina and optic nerve in MS; thus, the correlation between the two remains indirect. Indeed, it may be some time before sufficient postmortem samples with recent high resolution OCTs become available. A magnetic resonance imaging (MRI)/OCT study of the optic nerve and RNFL in MS perhaps is the closest to establishing this correlation, yet it was acknowledged that demyelination and axon loss could have been the substrate for the MRI evidence of optic nerve atrophy.^5^ It is clear that optic neuritis (ON) is a key event that precedes RNFL atrophy in many MS cases. However, it has been clearly shown that atrophy frequently occurs in the retina of patients without ON, albeit to a lesser degree.^1,27–29^ In a study of the optic nerve pathology from 18 MS patients, 35 of 36 nerves showed areas of demyelination, yet only 8 patients had a history of ON.^30^ Thus, the optic nerve frequently is involved in MS; hence, the ability to potentially monitor axon loss in the nerve is extremely important.

The moderate thinning of the RNFL of the *shp* (13.2 μm average loss) showed by sdOCT is in the range reported in MS (20 μm average loss; range, 5–40 μm).^31^ Noticeably there was morphologic evidence of marked retinal gliosis and Müller cell hyperplasia accompanying the loss of axons in the RNFL of *shp* dogs, and we believe that, if not for this reactive retinal glial response, the decrease in RNFL thickness would have been even more accentuated. As in MS, there were regional variations in the retinal atrophy in the *shp*, with the superior quadrant most noticeably affected, while in MS, the temporal retina is the primary site of atrophy.^32,33^ Studies on human patients with demyelinating disorders also have reported changes in other retinal layers beside the RNFL, including the bipolar cell layer. Syc et al.^34^ reported larger C/D ratios and decreased NRT in a large study of MS patients, as we also found in the *shp* dogs. In the *shp* animals, the C/D and NRT changes were present at an early age and were stable or changed very slowly over time.

The reason why atrophy of the RNFL is not uniform in MS and in the *shp* is not known but may relate to the size of axons that degenerate in the optic nerve. Gelfand et al.^35^ noted that affected areas of the retina in MS contained axons derived from small parvocellular neurons that have small diameter axons. In the optic nerve of all species where axon distribution has been quantitated, including man,^36^ nonhuman primate,^37^ and canine,^38^ the distribution of large and small myelinated axons is uneven throughout the nerve though small diameter axons are more prevalent in the core of the nerve. It is known that small diameter axons are preferentially involved in MS;^39^ hence, the patchy distribution of axon loss in the *shp* optic nerve report here may lead to more focal loss of small diameter axons in the RNFL. The longitudinal data on the four optic nerves of the *shp* dogs suggested there is progression of RNFL atrophy over time. Sequential scans (Figs. 2F–J) indicated that between 5 and 11 months there is relative increase in RNFL thickness of the superior quadrant, as a result of normal retinal development, but then there is subsequent atrophy. Also, the canine eye differs from the human in that it contains a tapetum in the superior hemiretina that may have an as yet undetermined role in RNFL loss in the superior quadrant in *shp*.

In MS, it has been reported that following a bout of ON, the RNFL can increase in thickness for a short period followed by subsequent thinning^40,41^ with relative stabilization for 7 to 12 months after acute disease. However, another large study showed progression of RNFL atrophy with time, even in patients without prior ON.^28^ The present study was not designed to determine whether there was progressive RNFL thinning in the *shp* dogs as that would require further longitudinal observations on more animals.

The mechanism of atrophy of the RNFL following visual pathway lesions in MS is incompletely understood. It has been postulated that it could result from direct loss of the neuronal cell body (RGCs), from retrograde degeneration of axons following their loss in the optic nerve, or from the subsequent atrophy of the proximal optic nerve axons in the RNFL following focal optic nerve axon loss.^42^ In our model, it appears that the thinning of the RNFL relates solely to loss of axons in the optic nerve, which results in retrograde degeneration. There was no quantitative evidence of atrophy of axons in the RNFL but there was a clear ultrastructural difference in the mutant compared to control dogs as seen on TEM and in the percentage of area of the RNFL occupied by axons. Retinal ganglion cells were clearly seen in 1 μm sections and present in normal numbers with no evidence of chromatolysis or apoptosis. In contrast, in MS there frequently but not always is a loss of RGCs and potentially other retinal neurons.^8^ Recent studies have demonstrated thinning of OCT-derived measurements of the RGCL on the macular and peripapillary regions of MS patients.^43^ Indeed, a subset of MS patients have been shown to have primary retinal pathology leading to RNFL thinning and not resulting from retrograde degeneration in the optic nerve.^44^ While the lack of a decrease in the RGC numbers observed here might be surprising, it may be due to retinal tissue sampling restrictions and to differences in the topographic distribution of RGCs in the dog retina. The dog retina presents a fovea-like area (area centralis) in a topographic area comparable to the human macula that has an increase in RGC and cone photoreceptor density.^45^ Despite these similarities the RGC concentration and total size of the area centralis are significantly smaller than the human fovea centralis,^46,47^ making it hard to adequately sample both histologically and by OCT. Also, the RGC counts in our study were obtained from the 1-μm cross-sections, and despite having sampled all retinal quadrants it is likely that we were not able to identify a subtle loss in RGCs.

In the *shp* dogs, ERGs recorded normal A and B waves suggesting that photoreceptors and bipolar cells are generating appropriate light-evoked responses. The PhNR, reduced in MS, with and without ON,^48^ also was reduced in the *shp* dogs, suggesting that remaining RGCs may have functional deficits. It is worth noting that VEPs are thought by some to be a more sensitive measure of optic nerve damage than RNFL thinning.^49–51^

We also recorded VEPs as an in vivo measure of axon function in the optic nerve. The aberrant fVEP response in *shp* dogs is consistent with abnormal VEPs reported in demyelinating diseases^12,52^ and also in PMD.^53^ The VEPs measured in this study were elicited by flash stimulation. Studies using multifocal pattern stimulation have shown a VEP amplitude reduction precedes axon loss in patients after ON.^54^ The flash-evoked VEPs from the *shp* were abnormal from the first tests conducted as would be expected given the severe dysmyelination of the nerve. The fact that the amplitude of the evoked response was much reduced could relate to RGC dysfunction, failed conduction along many axons due to lack of myelin, or to axon loss.

How relevant are these studies of sdOCT identification of RNFL thinning in an animal model of PMD to what has been reported in MS? A recent study on the retina of a murine model of PMD (the *jimpy* mouse) showed that the structure of the retina is normal at 22 days of age despite severe dysmyelination of the optic nerve though no loss of axons.^55^ The optic nerves in the *shp* show a clear loss of axons that appears to increase in time. The earliest evidence of axon degeneration was noted at 4 months, though notable areas of axon loss and gliosis were not apparent until much later (two or more years). We propose that this chronic loss of axons may resemble what happens in progressive MS, either primary or secondary, at least in a background of unensheathed axons with no or minimal inflammation.^56^ There is no evidence of inflammation in the *shp* optic nerves, brain, and spinal cord^16^ and, more recently, an exploration of differential gene expression in the spinal cord using RNAseq has shown no evidence of an increase in expression of inflammatory molecules (Duncan and Svaren, unpublished data). It is not known why optic nerve axons degenerate in this model, unlike axons in the brain and spinal cord that appear relatively well preserved.^16^ Perhaps the chronic lack of myelin insulation results in axon death due to a lack of growth factors, similar to what has been proposed to occur in chronic MS plaques^56,57^ and that this risk is greater for some reason in the optic nerve. Certainly, there are clear axon–glial–myelin sheath interactions that are required for long-term axon survival.^58–60^ Despite the fact that the relationship between optic nerve damage and RNFL thinning has been demonstrated in animal models of experimental glaucoma,^9,10^ the cellular mechanisms involved in this process are poorly understood. The *shp* model replicates what occurs following chronic myelin loss or absence and, hence, better matches what happens in MS.

It is clear that in future clinical trials of neuroprotection in MS, the ability to accurately monitor axon loss and survival is essential. Optical coherence tomography as a surrogate marker of axon loss in the optic nerve would appear, from the data presented here, to have a solid scientific basis. However, in the future, as a method of quantifying neuroprotection of optic nerve axons, it may be necessary to discern between those cases where the loss of axons in the RNFL with its subsequent atrophy is due to focal lesions in the optic nerve or a primary loss of RGCs.
